# Isoflavones from Semen Sojae Preparatum Improve Atherosclerosis and Oxidative Stress by Modulating Nrf2 Signaling Pathway through Estrogen-Like Effects

**DOI:** 10.1155/2022/4242099

**Published:** 2022-04-07

**Authors:** Jinzhou Guo, Jingxin Ma, Kun Cai, Haining Chen, Ke Xie, Binren Xu, Desen Quan, Jingyan Du

**Affiliations:** ^1^Guizhou University of Traditional Chinese Medicine, Gui Yang 550025, China; ^2^Liaocheng Women and Children Hospital, Liaocheng 252000, China; ^3^Jiangxi College of Traditional Chinese Medicine, Fuzhou 344000, China; ^4^Endocrine Metabolism Center of Taiyuan Central Hospital, Taiyuan 030000, China

## Abstract

Atherosclerosis (AS) often occurs in cardiovascular disease, which is a chronic vascular disease and is harmful to human health. Oxidative stress is involved in its etiology. This study aimed to determine the effectiveness of Isoflavones from semen sojae preparatum (ISSP) in inhibiting oxidative stress and its important molecular mechanisms through in vivo and in vitro experiments. ApoE^−/−^ mice were used to establish atherosclerosis models through a high-fat diet, and endothelial cells were used to establish oxidative stress injury models through ox-LDL induction. The degree of oxidative stress damage was assessed by detecting changes in ET-1, LDH, SOD, and MDA indicators. It was observed that after ISSP treatment, the oxidative stress damage of mice and endothelial cells was improved. The Nrf2/AER signaling pathway is an important antioxidant pathway that has attracted our attention. Western blotting and qRT-PCR were used to detect the expression of Nrf2, HO-1, and NQO1 in mice aortae and endothelial cells. The results showed that the Nrf2 signaling pathway was activated after ISSP intervention. In addition, in this study, after preantagonizing the estrogen receptors GPR30 and ER*β*, it was observed that the effects of ISSP in treating endothelial cell oxidative damage and activating the Nrf2 signaling pathway were weakened. After silencing Nrf2 by Nrf2-siRNA transfection, the effect of ISSP in treating endothelial cell oxidative damage was inhibited. This study shows that ISSP may reduce oxidative stress damage and atherosclerosis through the Nrf2 signaling pathway, and this effect may involve the GPR30 and ER*β* estrogen receptors.

## 1. Introduction

Atherosclerosis (AS) is formed from pathological products such as lipid accumulation in arteries, fibrosis, and atherosclerotic plaques in the arteries and then it causes chronic arterial vascular disease that weakens arterial elasticity and narrows the arterial cavity [1]. AS often occurs in the heart, brain, kidney, and other important organs, causing serious harm to human health. Epidemiological studies have shown that postmenopausal AS has an increased incidence in women [2], which is associated with diminished vasoprotective effects of endogenous estrogens. On this basis, estrogen replacement therapy is derived, but long-term estrogen use will cause many adverse reactions [3,4] and will increase the incidence of female malignant tumors [5].

Phytoestrogens are plant derivatives similar in structure and mode of action to 17-beta-estradiol, in which isoflavones are one of its main groups. Semen sojae preparatum (SSP) is fermented from soybeans, and its main active ingredient is isoflavones. At present, the antioxidation [6] and anti-AS [7] effects of phytoestrogens have been continuously studied and confirmed. Studies have found that phytoestrogens can reduce the formation of lipid peroxides [8] and alleviate the body damage caused by lipid peroxidation. They can inhibit LDL oxidation by changing the size of LDL particles, reduce the production of lipid peroxidation products, including MDA, and inhibit the occurrence of oxidative stress and damage to the vessel wall from oxidized lipids [9]. They can also increase antioxidant capacity by increasing levels of numerous antioxidant enzymes, including SOD and glutathione.

As one of the main coordinators of the oxidative stress response, nuclear E2-related factor 2 (Nrf2) is a type of nuclear transcription factor widely present in oxygen-consuming organs [10]. It can regulate the activities of various antioxidant enzymes [11]. Under physiological conditions, Nrf2 is immobilized in the cytoplasm [12]. When oxidative stress occurs, Nrf2 is released and enters the nucleus [13]. Nrf2 binds with AREs and directly activates its own transcription [14], thus providing a positive feedback mechanism to amplify the effect of Nrf2, and the activities of its downstream antioxidant enzymes are upregulated [15].

In this study, we explored the effects and mechanisms of ISSP on AS and oxidative stress, and attempted to elucidate the role of ISSP estrogen-like effects in this, and demonstrated the role of the Nrf2 signaling pathway.

## 2. Materials and Methods

### 2.1. Main Reagent

The following were used: Oil Red O solution, HE Staining Kit and Masson's Trichrome Stain Kit (Solarbio, Beijing, China), Lipofectamine™ 3000 reagent (Invitrogen, USA), Opti-MEM medium (Gibco, USA), G-15, MPP, and PHTPP (Cayman Chemical, USA), CCK-8 assay (Dojindo, Japan), Mouse ET-1 and ox-LDL ELISA Kit (Enzyme Immune, Jiangsu, China); Human ET-1 ELISA Kit (Elabscience, Wuhan, China), RIPA lysis buffer and BCA assay (Solarbio, Beijing, China), assay kits for LDH, TC, TG, HDL-C, LDL-C, SOD, and MDA (Jiancheng, Nanjing, China); antibodies against PCNA (Proteintech Group, Wuhan, China); *β*-actin (Absin, Shanghai, China); ER*α*, ER*β*, GPR30, and Nrf2 (Abcam, USA); HO-1 and NQO1 (CST, USA); 10% SDS–PAGE gels (Bio-Rad, USA), PVDF membranes (Millipore, MA, USA) and GeneJET PCR Purification Kit (Thermo Scientific, USA), PrimeScript™ RT reagent Kit with gDNA Eraser and TB Green® Premix Ex Taq™ II (Takara, Japan) was used.

### 2.2. Ethics Statement

The experimental process strictly followed the relevant laws and regulations of the Guizhou University of Traditional Chinese Medicine Animal Experiment Ethics Committee and passed the laboratory animal welfare and ethical review of Guizhou University of Traditional Chinese Medicine.

### 2.3. Preparation of Isoflavones from Semen Sojae Preparatum (ISSP)

Preparation of pure fermented tempeh extract: black soybeans were used as the raw materials, soaked in the decoction of *Artemisia annua* and mulberry leaves (1 g/L) for 12 hours, and then sterilized at 105°C for 15 minutes for use. The high-efficiency fermentation strain (CAK-05) selected by the research group in the early stage was inoculated into the black soybeans treated as above, cultured at 25°C for 9 days, and then removed and dried. The sample was crushed and subjected to petroleum ether degreasing, 80% ethanol ultrasonic extraction, and HPD100 macroporous resin purification treatment to obtain a light tempeh extract.

### 2.4. Animals and Treatmen*t*

ApoE^−/−^ female mice weighing 20−25 g were selected (Beijing Vital River Laboratory Animal Technology Co., Ltd.) and kept at 21−24°C, relative humidity 40−55%, and indoor light for 12 h, darkness for 12 h. The mice were allowed to drink and eat freely (provided with high-pressure sterilized water). After 1 week, ApoE^−/−^ female mice were treated with a sham operation or bilateral ovariectomy (OVX) and fed ordinary feed for one week after the operation. During this period, the mice were examined by vaginal smears, and OVX-operated mice with negative keratinocytes for 5 days were used as the experimental subjects. A random distribution method was applied, which divided the mice into six groups (*n* = 8 per group), and the intervention methods were as follows: sham group (sham + HFD), Mod group (OVX + HFD), AC group (OVX + HFD + AC 0.152 mg/kg), ISSP (H) group (OVX +HFD + ISSP 10 mg/kg), ISSP (M) group (OVX + HFD + ISSP 5 mg/kg), and ISSP (L) group (OVX + HFD + ISSP 2.5 mg/kg). The HFD intragastric volume was 0.1 ml/10 g, and all treatments lasted for ten weeks. Then, the mice were anesthetized with 10% pentobarbital sodium. Blood was taken from the eyeball veins and the aortic tissue was retained for subsequent analysis.

### 2.5. Cell Culture

EA.hy926 cells (Kunming Cell Bank, Kunming, China) were cultured using a serum-containing medium (containing 10% FBS, 90% DMEM/F-12 medium, and 1% penicillin/streptomycin). The cells were incubated in a 5% CO_2_ humidified 37 C atmosphere.

### 2.6. siRNA Transient Transfection

The siRNA was synthesized by Shanghai Gima Co., Ltd., and the sequences of the RNA oligos are shown in Table 1. EA. hy926 cells were cultured in 6-well plates with an Opti-MEM medium. EA.hy926 cells were transfected with LipofectamineTM 3000 reagent for 12 hours according to the manufacturer's instructions. The transfection reagent was discarded, and the cells were cultured in a standard growth medium.

### 2.7. Antagonize Estrogen Receptor

EA hy926 cells were pretreated with GPR30 receptor antagonist (G-15), ER*α* receptor antagonist (MPP), and ER*β* receptor antagonist (PHTPP) for 2 hours in advance [16, 17], then ox-LDL and ISSP were added, and the cells were incubated for 24 hours.

### 2.8. Blood Lipid Level Assessment

To collect serum, the levels of TC, TG, HDL-C, and LDL-C were measured with commercial kits according to the manufacturer's instructions.

### 2.9. Oil Red O, H&E, and Masson Staining

After removing excess tissues around the aorta, it was cut longitudinally with microscissors and stained with Oil Red O. The paraffin embedding method was used for the mouse aortic arch, and the cryo-embedding method was used for the mouse aortic sinus. The thicknesses of the paraffin sections and frozen sections were 4 *μ*m and 7 *μ*m, respectively. Frozen sections of the aortic sinus were used for Oil Red staining, HE staining, and Masson staining. Paraffin sections of the aortic arch were used for HE staining and Masson staining. The experiment was completed according to the manufacturer's instructions. The sections were viewed and recorded under an optical microscope.

### 2.10. Assessment of Cell Viability

The cells were seeded in 96-well plates at 1 × 10^4^ cells/well, and cultured for 22 h. After discarding the culture supernatant, 100 *μ*l of reagent (containing 90% DMEM/F12 medium and 10% CCK-8 assay) was added and the incubation was continued for 2 h, and the optical density was measured at 450 nm.

### 2.11. LDH Analysis

Cell culture supernatants and serum were collected. The LDH levels were tested according to the manufacturer's instructions for the lactate dehydrogenase assay kit.

### 2.12. ELISA

To collect serum and cell culture supernatant, the levels of ox-LDL and ET-1 in serum and the levels of ET-1 in the cell culture supernatant were detected according to the commercial ELISA kit's instructions.

### 2.13. Determination of SOD and MDA

Cells/tissues lysates were prepared with RIPA lysis buffer, and the protein concentration in the lysates was measured by BCA assay. SOD and MDA were measured with commercial kits according to the manufacturer's instructions.

### 2.14. Western Blot Analysis

In brief, the total protein of cells/tissues was prepared with RIPA lysis buffer, and the nucleoprotein of the cell/tissue was prepared with a Nuclear Protein Extraction Kit. After that, the protein concentrations were determined by BCA assay, and the proteins were separated electrophoretically by 10% SDS–PAGE before being transferred to PVDF membranes. Then, 5% skimmed milk was used to block the membrane before incubation with specific primary antibodies overnight at 4°C against PCNA (1 : 1000), *β*-actin (1 : 1000), ER*α* (1 : 1000), ER*β* (1 : 1000), GPR30 (1 : 250), Nrf2 : 2000), HO-1 (1 : 1000), and NQO1 (1 : 1000). Afterwards, the membranes were incubated with the secondary antibodies at room temperature for 1 h. They were densitometrically quantified with ImageJ software.

### 2.15. qRT-PCR Analysi*s*

Total RNA was extracted from the cells and tissues with a GeneJET PCR Purification Kit. cDNA was synthesized using the PrimeScript™ RT Reagent Kit with gDNA Eraser. TB Green® Premix Ex Taq™ II and CFX96 Real-Time PCR Detection System were used to perform qRT-PCR. The samples were analyzed in triplicate, and GAPDH or *β*-actin served as internal controls. Primer sequences of the target genes are shown in Table 2.

### 2.16. Statistical Analysis

Data analysis and image production were carried out using SPSS 19.0 and GraphPad Prism 7.0 software. The data are expressed as $\bar{x}$ ± *s*. The comparison between multiple groups was performed using one-way ANOVA, and the pairwise comparison was performed using LSD. The comparison between the two groups was analyzed by Student's *t*-test; *P* < 0.05 was considered statistically significant.

## 3. Results

### 3.1. ISSP Ameliorated Atherosclerosis and Altered Blood Lipid Homeostasis in HFD-OVX-Treated ApoE^−/−^ Mice

**Figure 1:**
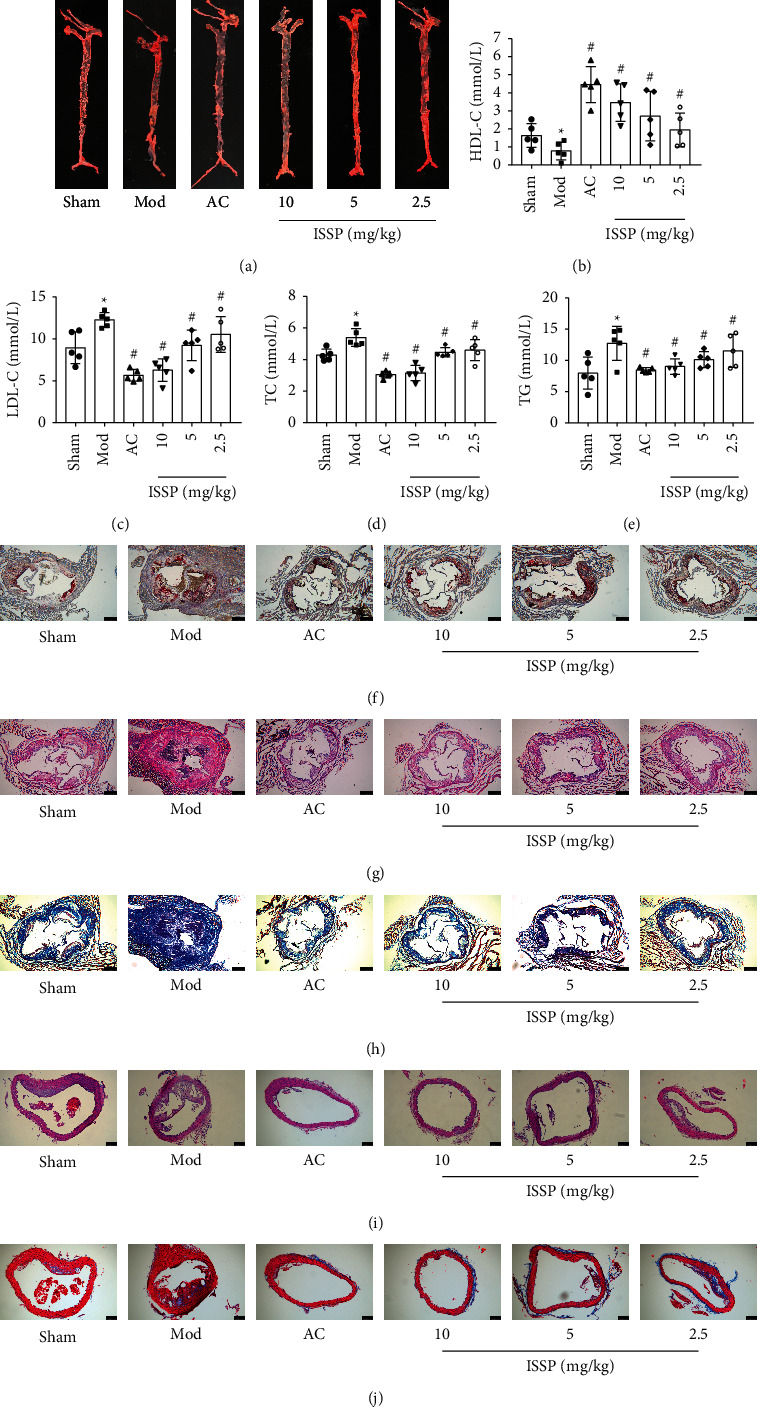
ISSP ameliorated atherosclerosis and altered blood lipid homeostasis in HFD-OVX-treated ApoE^−/−^ mice. (a) The gross Oil Red O staining for aortic plaque in ApoE^−/−^ mice. (b) The level of HDL-C. (c) The level of LDL-C. (d) The level of TC. (e) The level of TG. (f) Oil Red O staining on aortic sinus. (g) H&E staining on aortic. (h) Masson staining on aortic. (i) H&E staining on the aortic sinus. (j) Masson staining on the aortic sinus. The data are denoted as mean ± SD (*n* = 5), ^∗^*P* < 0.05 indicates a significant difference compared with the sham group; ^#^*P* < 0.05 indicates a significant difference compared with the Mod group.

Frozen sections of the entire aorta and aortic sinus were stained with Oil Red O to evaluate AS. As shown in Figures 1(a) and 1(f), the Oil Red O staining lesions in the model group were significantly larger than those in the sham operation group, and the ISSP intervention improved the aortic lesions. At the same time, as shown in Figures 1(b)–1(e), the level of HDL-C in the model group was significantly lower than that in the sham operation group, while the levels of LDL-C, TC, and TG were significantly higher. In contrast, after ISSP intervention, the HDL-C levels were increased, and the LDL-C, TC, and TG levels were reduced, which was significantly different from the model group. Next, H&E staining and Masson staining were used to evaluate the plaque area and stability of the aorta and aortic sinus. As shown in Figures 1(g)–1(j), the plaque area of the model group was greater than that of the sham group, and the collagen content and unstable plaque were reduced in the model group. After ISSP intervention, the plaque area and stability were significantly improved. The above results are more obvious when using higher ISSP concentrations.

### 3.2. ISSP Attenuates Oxidative Damage and Activated the Nrf2 Signaling Pathway in HFD-OVX-Treated ApoE^−/−^ Mice

**Figure 2:**
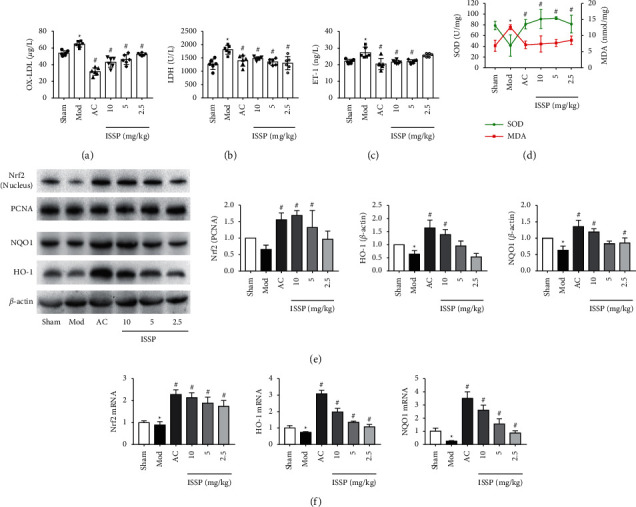
ISSP attenuated oxidative damage and activated the Nrf2 signaling pathway in HFD-OVX-treated ApoE^−/−^ mice. (a) The level of ox-LDL. (b) The level of LDH. (c) The level of ET-1. (d) The level of SOD and MDA. (e) The western blot examined protein levels. (f) The mRNA expression level of Nrf2, HO-1, and NQO1 mRNA. The data are denoted as mean ± SD (a-d, *n* = 6; e and f *n* = 3). ^*∗*^*P* < 0.05indicates a significant difference compared with the sham group; on the other hand, compared with the Mod group, ^#^*P* < 0.05indicates that there is a statistically significant difference.

The oxidative damage of ApoE^−/-^ mice is shown in Figures 2(a)–2(d), the levels of ox-LDL, LDH, and ET-1 in the model group were increased and were significantly different from those in the sham operation group, and ISSP intervention ameliorated their increase. The SOD and MDA test results showed that the SOD activity of the model group decreased; in contrast, the MDA content increased, which was significantly different from that of the sham operation group. After ISSP treatment, increased SOD activity and decreased MDA content were significantly different from those of the model group. ISSP intervention showed the opposite.

The protein expression results showed that Nrf2 (nucleus), HO-1, and NQO1 protein expression was increased with ISSP intervention (10 mg/kg) (Figure 2(e)). Similar to the protein expression, the expression of Nrf2, HO-1, and NQO1 mRNA was also increased with ISSP intervention (10 mg/kg) (Figure 2(f)). These results were significantly different from the model group.

### 3.3. ISSP Attenuates Oxidative Damage and Activated the Nrf2 Signaling Pathway of EA.hy926 Cells Treated with Ox-LDL

**Figure 3:**
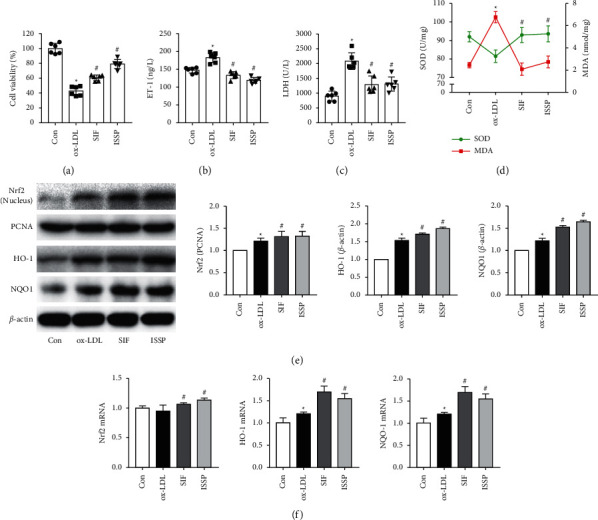
ISSP attenuated oxidative damage and activated the Nrf2 signaling pathway of EA.hy926 cells treated with ox-LDL. (a) The cell viability assessment. (b) The level of ET-1. (c) The level of LDH. (d) The level of SOD and MDA. (e) The western blot examined protein levels. (f) The expression level of Nrf2 mRNA, HO-1 mRNA, and NQO1 mRNA. The data are denoted as mean ± SD (a–d *n* = 6; e and f, *n* = 3). ^*∗*^*P* < 0.05indicates a significant difference compared with the control group; on the other hand, compared with the ox-LDL intervention group, ^#^*P* < 0.05indicates that there is a statistically significant difference.

Previous studies have shown that ox-LDL plays a key role in the occurrence and development of atherosclerotic oxidative damage. Therefore, ox-LDL was chosen as a drug to induce EA.hy926 cell oxidative damage. We first evaluated the impact of ISSP on oxidative damage in endothelial cells. The results are shown in Figures 3(a)–3(d). The ox-LDL intervention reduced the cell viability and SOD activity. In contrast, the ET-1 level, LDH activity, and MDA content increased, and there was a significant difference compared with the control group. This shows that the oxidative damage model was successfully prepared after the ox-LDL intervention. Subsequently, ISSP intervention ameliorated ox-LDL-induced oxidative damage to endothelial cells, which is consistent with the results of previous in vivo experiments.

We detected the protein and mRNA expression levels in the Nrf2 signaling pathway in endothelial cells. We observed that under normal circumstances, the protein expression level of Nrf2 (nucleus) was low. After the ox-LDL intervention, the protein expression of Nrf2 (nucleus), HO-1, and NQO1 was significantly upregulated. Similarly, the expression of HO-1 and NQO1 mRNA was significantly upregulated after the ox-LDL intervention, but it did not cause significant changes in the expression of Nrf2 mRNA. Treatment with ISSP further upregulated their expression, which was significantly different from the model group (Figures 3(e) and 3(f)).

### 3.4. Antagonizing Estrogen Receptors Attenuates ISSP Treatment of Oxidative Damage and ISSP's Activation of Nrf2 Signaling Pathway in EA.hy926 Cells

**Figure 4:**
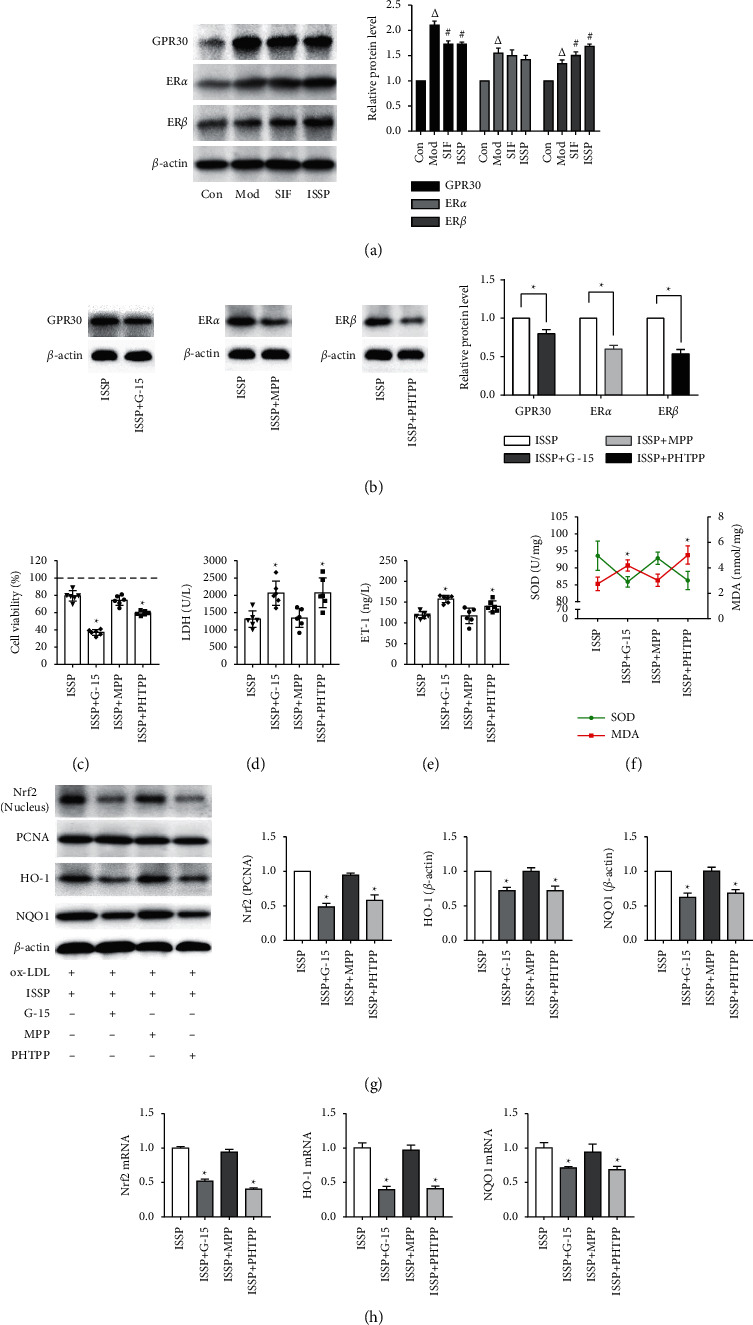
Antagonizing estrogen receptors attenuates ISSP treatment of oxidative damage and ISSP's activation of Nrf2 signaling pathway in EA.hy926 cells. (a) The western blot examined protein levels of estrogen receptors. (b) The expression level of estrogen receptors protein after preintervention with receptor-specific antagonists. (c) The cell viability assessment. (d) The level of LDH. (e) The level of ET-1. (f) The level of SOD and MDA. (g) The western blot examined protein levels. (h) The expression level of Nrf2 mRNA, HO-1 mRNA, and NQO1 mRNA. The data are denoted as mean ± SD (a, b, g and h, *n* = 3; c– f, *n* = 6). ^△^*P* < 0.05 indicates a significant difference compared with the control group; On the other hand, compared with the Mod group, #*P* < 0.05 indicates that there is a statistically significant difference and ^*∗*^*P* < 0.05 indicates a significant difference compared with the ISSP group.

ISSP is a type of phytoestrogen. Therefore, we speculated that the effect of ISSP in improving endothelial cell oxidative damage may depend on its estrogen-like effect. To verify this conjecture and further explore the mechanism of ISSP, we used an estrogen receptor antagonist with 2 hours of pretreatment and then intervened with ISSP and ox-LDL. Under ox-LDL stimulation, the expression of the ER*α*, ER*β*, and GPR30 proteins in EA.hy926 cells was increased. Compared with the model group, after ISSP intervention in the cells, there was no significant difference in ER*α* protein expression, GPR30 protein expression was decreased, and ER*β* protein expression was increased (Figure 4(a)). Compared with ISSP intervention, preintervention with ER*α*, ER*β*, and GPR30 receptor-specific antagonists reduced the expression of each estrogen receptor protein (Figure 4(b)).

Compared with the ISSP group, after preantagonizing, ER*β* and GPR30 receptors significantly increased the LDH activity, ET-1 level, MDA content. In contrast, the cell viability and the SOD activity were decreased. There was no significant change in the degree of cell oxidative damage after preantagonizing ER*β* (Figures 4(c)–4(f). The protein and mRNA expression results are shown in Figures 4(g)–4(h). Compared with the ISSP group, the preantagonizing ER*β* and GPR30 receptors significantly upregulated the expression of Nrf2 (nucleus) HO-1 and NQO1. The mRNA expression results were consistent with the protein results. After preantagonizing the ER*α* receptor, the levels of the proteins and mRNA did not change significantly.

### 3.5. Cell Transfection Effect and the Effect of Silencing Nrf2 Gene on Nrf2 Signaling Pathway and Endothelial Cell Oxidative Damage

**Figure 5:**
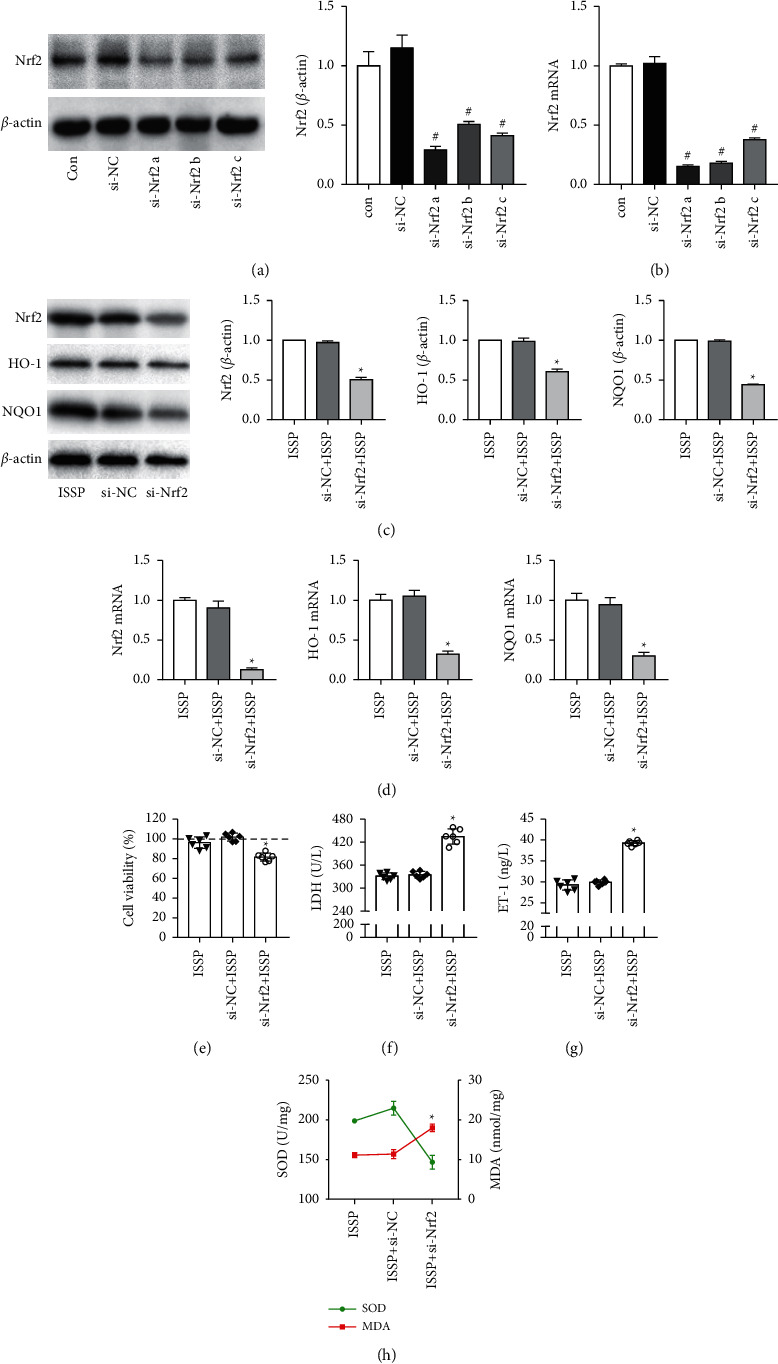
Cell transfection effect and the effect of silencing Nrf2 gene on Nrf2 signaling pathway and endothelial cell oxidative damage. (a) The western blot examined protein levels of Nrf2. (b)The expression level of Nrf2 mRNA in cells transfected. (c) The western blot examined protein levels. (d) The expression level of Nrf2 mRNA, HO-1 mRNA, and NQO1 mRNA. (e) The cell viability assessment. (f) The level of LDH. (g) The level of ET-1. (h) The level of SOD and MDA. The data are denoted as mean ± SD (a–d, *n* = 3; e–h, *n* = 6). ^#^*P* < 0.05 indicates a significant difference compared with the si-NC group; on the other hand, compared with the si-NC + ISSP group, ^*∗*^*P* < 0.05 indicates that there is a statistically significant difference.

As shown in Figures 5(a)–5(b), forty-eight hours after the cells were transfected, the protein and mRNA expression levels of Nrf2 in the si-Nrf2a, si-Nrf2b, and si-Nrf2c groups were significantly lower than those in the si-NC group, and the si-Nrf2a group had the lowest expression levels. Therefore, this siRNA was used in the following gene silencing experiments.

In order to confirm the role of Nrf2 in ISSP's reduction of endothelial cell oxidative damage, after silencing the Nrf2 gene, we detected the expression of Nrf2 signaling pathway-related proteins and mRNA and evaluated its effect on endothelial cell oxidative damage. The experimental results are shown in Figures 5(c)–5(h). The LDH activity, ET-1 level, MDA content in the si-Nrf2 group were increased. The cell viability, SOD activity, and the protein and mRNA expression levels of Nrf2, HO-1, and NQO1 in the si-Nrf2 group were decreased; and there was a significant difference compared with the si-NC group. These results indicated that the effect of ISSP in improving endothelial cell oxidative damage was inhibited after blocking the Nrf2 signaling pathway.

## 4. Discussion

Studies have shown that the incidence of AS in postmenopausal women is higher than that before menopause, which is closely related to the loss of endogenous estrogen protection on the vascular endothelium [18]. Our experimental results showed that ovariectomy significantly aggravated the degree of AS in mice. Oxidative stress is one of the important reasons for the occurrence and development of AS. Oxidative stress damage is caused by the imbalance of the oxidation/antioxidant system in the body and is then aggravated by a series of damages to the body. When oxidative stress occurs, the ability of antioxidant enzymes in the body to remove oxidative substances is relatively weakened, and the accumulation of reactive oxygen species (ROS) in the body increases, causing body damage [19]. Oxidative stress can also cause a variety of damages, including cell lipid peroxidation, leading to oxidative damage to the blood vessel wall [20]. Therefore, oxidative stress is one of the important reasons for the occurrence and development of AS. Our experimental results showed that the occurrence of AS in mice is accompanied by the occurrence of oxidative stress damage. At the same time, the AS and oxidative damage were improved after ISSP intervention. These results suggest that ISSP may reduce the occurrence of atherosclerosis and oxidative stress in mice through estrogen-like effects.

It is worth mentioning that our experimental results showed that with the occurrence of atherosclerosis and oxidative stress in mice, the level of ox-LDL in mice also increases. The previous view was that oxidative stress is closely related to ox-LDL [21]. Previous studies have suggested that LDL initiates the production of a large number of unsaturated fatty acids, and oxidation allows them to form ox-LDL, which initiates and accelerates AS, as the key to the formation of lesions [22]. The formation of foam cells in the development of AS is its most important pathological feature, and it plays an important role in all stages of its development [23, 24]. Macrophages recognize and engulf ox-LDL bound to the scavenger receptor on its surface and then form foam cells. At the same time, the cytotoxic effect of ox-LDL also promotes the formation of foam cells [25]. In many previous studies, ox-LDL was used as a modeling drug to induce oxidative damage to endothelial cells [26–28]. Therefore, in further research on the mechanism of ISSP, ox-LDL was used as a model drug to induce oxidative damage in endothelial cells. Our in vitro experimental results show that ox-LDL intervention can induce endothelial cell oxidative damage. This is consistent with the results of in vivo experiments. ISSP intervention improved endothelial cell oxidative damage induced by ox-LDL and further showed that ISSP can improve the body's oxidative damage.

SSP is a product fermented with soybean as a substrate, and its effective drug ingredient isoflavone is a type of phytoestrogen. We mentioned earlier that the loss of endogenous estrogen protection from the body's vascular endothelium aggravated the onset of AS. Our experimental results also showed that the degree of AS and oxidative stress in mice is more pronounced after ovariectomy. Thus, we believe that ISSP reduces the occurrence of oxidative damage and AS, which may be related to its estrogenic effects. Estrogen receptors include nuclear receptors and membrane receptors. Among them, the nuclear receptors are ER*α* and ER*β* [29]. It has been reported that soybean isoflavones can activate ER*β* but have no effect on ER*α* [30]. The membrane receptors are currently widely recognized as GPR30, and there is evidence that GPR30 has cardioprotective effects [31] and has a preventive effect against atherosclerosis [32]. The results of our experiment showed that compared with the model group, after the intervention of ISSP, the expression of ER*β* protein was increased, and the expression of GPR30 protein was decreased. After preintervention with estrogen receptor antagonists, the protein expressions of the three estrogen receptors were significantly reduced. Next, after preintervention with a specific estrogen receptor antagonist, the effect of ISSP on improving oxidative damage was evaluated. The experimental results showed that after antagonizing the receptor ER*β* and the receptor GPR30, the effect of ISSP attenuates oxidative damage of EA.hy926 cells treated with ox-LDL was inhibited. After antagonizing the nuclear receptor ER*α*, the effect of ISSP was not inhibited. Unfortunately, due to the effect of ox-LDL on the expression of three estrogen receptor proteins, this experiment failed to clarify the effect of ISSP on estrogen receptors, but these results at least prove that ISSP ameliorates oxidative stress damage through estrogen receptor ER*β* and GPR30.

The Nrf2 signaling pathway is well known as an important antioxidant pathway. Studies have shown that Nrf2 is one of the main coordinators of oxidative stress and it belongs to the Cap'n'Collar (CNC) subfamily [33], which is a type of nuclear transcription factor with a bZIP structure in the basic region that is widely found in oxygen-consuming organs [34]. Under physiological conditions, Nrf2 is fixed in the cytoplasm [12] and cannot enter the nucleus to play a regulatory role. When oxidative stress occurs, Nrf2 is released [13] and it can smoothly enter the nucleus, bind to the AREs [35], and ultimately affect the activities of various antioxidant enzymes [36], activating various signaling pathways. Therefore, we speculate that the Nrf2 signaling pathway may be the focus of our next research. Subsequently, we tested the key factor proteins and mRNAs of the mouse aortic and endothelial cell Nrf2 signaling pathways. The results of this study show that ISSP can effectively activate the Nrf2 signaling pathway, and after antagonizing the nuclear receptor ER*β* and membrane receptor GPR30, the activation of the Nrf2 signaling pathway by ISSP is inhibited. To further prove its role in improving the oxidative stress of ISSP, we silenced the Nrf2 gene and observed its effect on ISSP. The experimental results showed that after silencing the Nrf2 gene and blocking the Nrf2 signaling pathway, the effect of ISSP in improving endothelial cell oxidative damage was significantly reduced, which indicated that it is an important pathway of ISSP.

## 5. Conclusion

**Figure 6:**
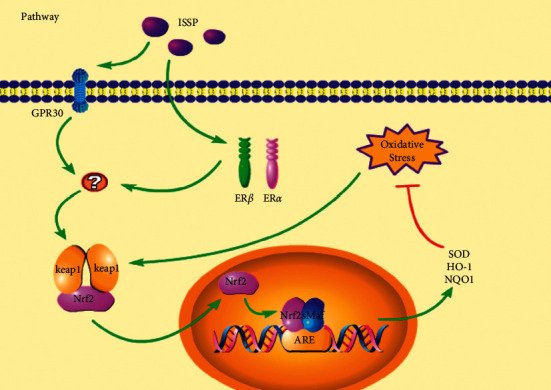
Summary and speculation on the potential mechanism of ISSP in improving oxidative stress based on the results of this experiment.

In conclusion, this study demonstrated the effect of ISSP in improving oxidative stress damage in vivo and in vitro for the first time and to explore the effect of ISSP estrogen-like effects on oxidative stress, as well as the role played by the Nrf2 signaling pathway. Our experimental results demonstrate that ISSP may exert estrogen-like effects through ER*β* and GPR30 receptors and further regulate the Nrf2 signaling pathway to improve oxidative stress (Figure 6).

## Figures and Tables

**Table 1:** siRNA base sequence.

Gene	Base sequence
si-Nrf2 a	(Sense) 5′-CCAGAACACUCAGUGGAAUTT-3′
	(Antisense) 5′-AUUCCACUGAGUGUUCUGGTT-3′
si-Nrf2 b	(Sense) 5′-GACAGAAGUUGACAAUUAUTT-3′
	(Antisense) 5′-AUAAUUGUCAACUUCUGUCTT-3′
si-Nrf2 c	(Sense) 5′-GGUUGAGACUACCAUGGUUTT-3′
	(Antisense) 5′-AACCAUGGUAGUCUCAACCTT-3′
si-NC	(Sense) 5′-UUCUCCGAACGUGUCACGUTT-3′
	(Antisense) 5′-ACGUGACACGUUCGGAGAATT-3′

**Table 2:** Primer sequences.

Gene	Endothelial cell	Gene	Mice
Nrf2	(F) 5′-TCCAGTCAGAAACCAGTGGAT-3′	Nrf2	(F) 5′-TCCAGTCAGAAACCAGTGGAT-3′
	(R) 5′-GAATGTCTGCGCCAAAAGCTG-3′		(R) 5′-GAATGTCTGCGCCAAAAGCTG-3′
HO-1	(F) 5′-AAGACTGCGTTCCTGCTCAACT-3′	HO-1	(F) 5′-AAGACTGCGTTCCTGCTCAACT-3′
	(R) 5′-AAAGCCCTACAGCAACTGTCG-3′		(R) 5′-AAAGCCCTACAGCAACTGTCG-3′
NQO1	(F) 5′-GAGAAGAGCCCTGATTGT-3′	NQO1	(F) 5′-GAGAAGAGCCCTGATTGT-3′
	(R) 5′-AAAGGACCGTTGTCGTAC-3′		(R) 5′-AAAGGACCGTTGTCGTAC-3′
GAPDH	(F) 5′-AGAAGGCTGGGGCTCATTTG-3′	*β*-actin	(F) 5′-AGAAGGCTGGGGCTCATTTG-3′
	(R) 5′-AGGGGCCATCCACAGTCTTC-3′		(R) 5′-AGGGGCCATCCACAGTCTTC-3′

## Data Availability

The data used to support the findings of this study may be obtained from the corresponding authors upon request.
